# Host Plant Selection Imprints Structure and Assembly of Fungal Community along the Soil-Root Continuum

**DOI:** 10.1128/msystems.00361-22

**Published:** 2022-08-09

**Authors:** Fengqiao Li, Zhili Jin, Zichen Wang, Yangwenke Liao, Li Yu, Xiaogang Li

**Affiliations:** a College of Biology and the Environment, Nanjing Forestry University, Nanjing, China; b Yongzhou Tobacco Company of Hunan Province, Yongzhou, Hunan, China; c Co-Innovation Center for Sustainable Forestry in Southern China, Nanjing Forestry University, Nanjing, China; Institute of soil science, Chinese academy of sciences

**Keywords:** rhizoplane, rhizosphere soil, fungal assembly, tobacco, compartment niche

## Abstract

The soil fungal community plays pivotal roles in soil nutrient cycling and plant health and productivity in agricultural ecosystems. However, the differential adaptability of soil fungi to different microenvironments (niches) is a bottleneck limiting their application in agriculture. Hence, the understanding of ecological processes that drive fungal microbiome assembly along the soil-root continuum is fundamental to harnessing the plant-associated microbiome for sustainable agriculture. Here, we investigated the factors that shape fungal community structure and assembly in three compartment niches (the bulk soil, rhizosphere, and rhizoplane) associated with tobacco (Nicotiana tabacum L.), with four soil types tested under controlled greenhouse conditions. Our results demonstrate that fungal community assembly along the soil-root continuum is governed by host plant rather than soil type and that soil chemical properties exert a negligible effect on the fungal community assembly in the rhizoplane. Fungal diversity and network complexity decreased in the order bulk soil > rhizosphere > rhizoplane, with a dramatic decrease in Ascomycota species number and abundance along the soil-root continuum. However, facilitations (positive interactions) were enhanced among fungal taxa in the rhizoplane niche. The rhizoplane supported species specialization with enrichment of some rare species, contributing to assimilative community assembly in the rhizoplane in all soil types. *Mortierella* and *Pyrenochaetopsis* were identified as important indicator genera of the soil-root microbiome continuum and good predictors of plant agronomic traits. The findings provide empirical evidence for host plant selection and enrichment/depletion processes of fungal microbiome assembly along the soil-root continuum.

**IMPORTANCE** Fungal community assembly along the soil-root continuum is shaped largely by the host plant rather than the soil type. This finding facilitates the implementations of fungi-associated biocontrol and growth-promoting for specific plants in agriculture practice, regardless of the impacts from variations in geographical environments. Furthermore, the depletion of complex ecological associations in the fungal community along the soil-root continuum and the enhancement of facilitations among rhizoplane-associated fungal taxa provide empirical evidence for the potential of community simplification as an approach to target the plant rhizoplane for specific applications. The identified indicators *Mortierella* and *Pyrenochaetopsis* along the soil-root microbiome continuum are good predictors of tobacco plant agronomic traits, which should be given attention when manipulating the root-associated microbiome.

## INTRODUCTION

Plants and microbes have been interacting with each other and evolving for their mutual benefit (1, 2). Consequently, the ability of root-associated microbiota (i.e., rhizomicrobiota) to facilitate the growth and health of the host plant via phytohormone production and competition with pathogens, respectively, is a subject of intense research (3–5). Furthermore, harnessing the root-associated microbiome is increasingly perceived as a sustainable approach to facilitate agricultural production (6, 7). Understanding of fundamental ecological processes that shape the microbiome assembly along the soil-root continuum is prerequisite for the precise manipulation of the microbiome in a specific niche.

The complexity of root-associated microbial communities is governed largely by the attractant and repellent activities of the host plant (8–10). The host root provides a nutrient-rich niche for microbes, in which the plant-microbe interactions are fostered by plant innate immunity (11). Meta-transcriptome analysis revealed differences in the bacterial microbiomes in the bulk soil and rhizosphere of several plant species (12), with a higher proportion of active bacteria associated with roots than with the bulk soil (13). Furthermore, bacterial and fungal communities associated with wheat “total roots” (i.e., including the endosphere and rhizoplane compartments) are clearly distinguished from those in the bulk soil and rhizosphere (14). Apart from the filtering effect of the host plant, the microbial community is also shaped by other factors, i.e., cropping practices, soil types, and nutrients (14–17). For instance, soil type is thought to account for early microbial community assembly in the plant rhizosphere (18–20), as the soil is a reservoir of diverse microbes. While, to date, studies have focused mainly on bacterial members of the overall microbial community, many issues remain concerning the fungal microbiome assembly by the host plant and in relation to the soil type in different niches (i.e., the bulk soil, rhizosphere, and rhizoplane) (14, 21, 22). Understanding of the ecological processes that shape the soil fungal community is essential, as fungi play important roles in the soil ecosystem, e.g., symbiosis (23, 24), nutrient cycling (25), decomposition (26, 27), pathogenesis (28, 29), and N_2_O production (29, 30). For instance, the versatile lifestyle and nutritional adaptability of *Trichoderma* enable several members of this genus to establish symbiotic interactions with the host plant, with *Trichoderma*-based products commercialized as biopesticides and biofertilizers (31–33).

Fungi appear to be more sensitive to microenvironmental variations than bacteria (34, 35) probably because most soil fungi are saprophytic and are constantly searching for available nutrients, e.g., by developing mycelia. Furthermore, symbiotic fungi aid plant nutrient absorption by forming mutualistic associations with plant roots, as the fungal hyphae extend beyond the area of nutrient depletion in root vicinity (36, 37) and grow in soil pores whose diameters are considerably smaller than that of the root to exploit nutrients (38). Additionally, some fungal species survive in a yeast form or as a mycelium depending on the environmental and some internal conditions, which facilitates their adaptation to the environment (39). Finally, different ecological niches impact the taxonomic groups and functions of the microbiomes within them (13, 22, 40).

Considering the above information, it is imperative to elucidate fluctuations in fungal communities along the soil-root continuum and to decipher the effects of host plant selection and soil variables therein. These data would add another dimension to the notion that host plant selection acts as a driver of microbial community variation along the soil-root continuum (11, 22). Accordingly, we established a greenhouse pot experiment involving four different soil types from major tobacco-producing areas in China and examined fungal community assembly in three distinct compartment niches therein (the bulk soil, rhizosphere, and rhizoplane). We hypothesized that (i) the contributions of plant selection and soil type to fungal community assembly along the soil-root continuum would shift across the bulk soil to the rhizosphere and the rhizoplane, (ii) the diversity and network complexity of the fungal community would decrease with an increasing plant selection effects, and (iii) the indicator taxa in the compartment niches would serve as a good predictor of plant agronomic traits.

## RESULTS

### Host plant shapes the fungal community assembly along the soil-root continuum to a greater extent than soil type.

**FIG 1:**
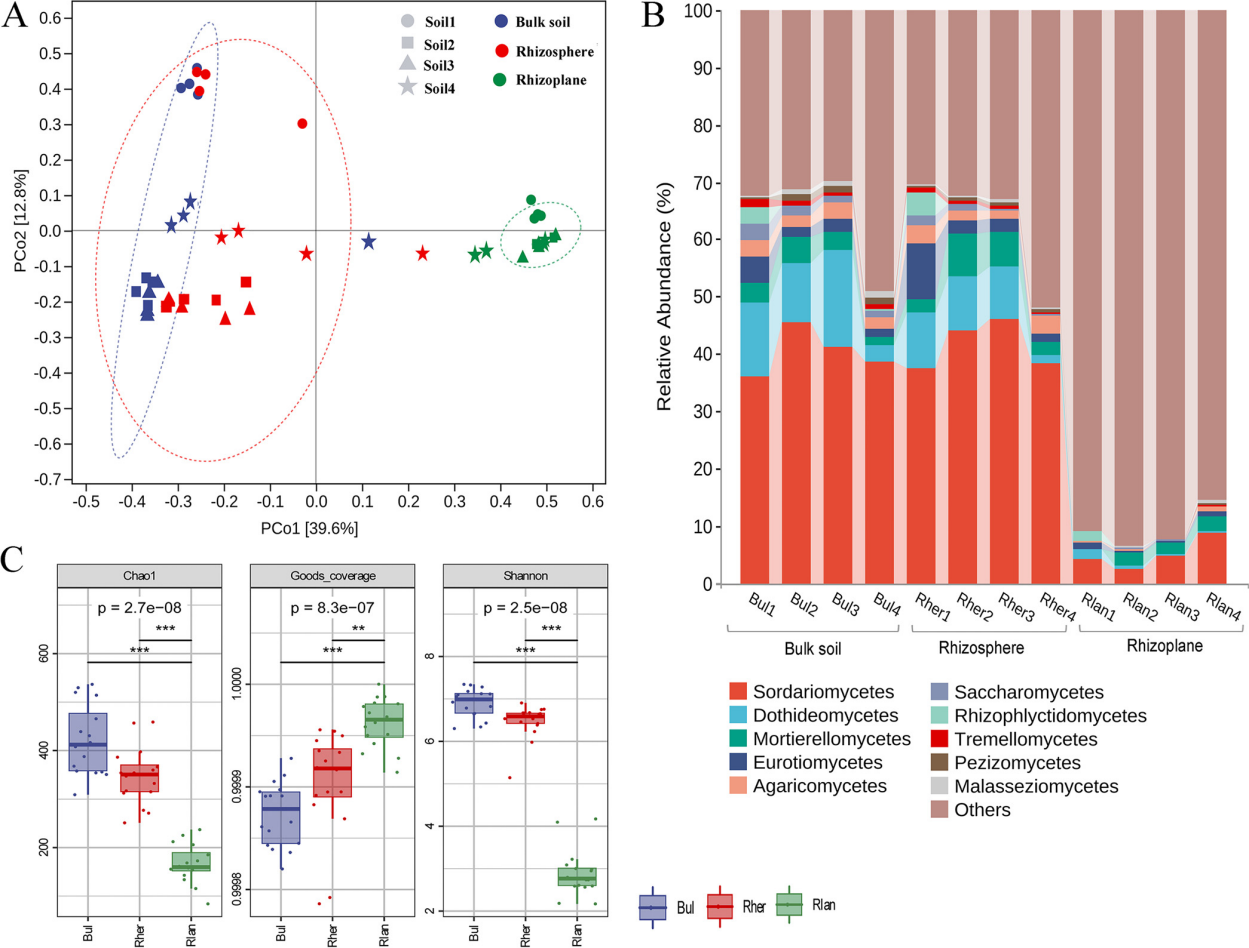
Host plant dominates over soil type in shaping the soil-root continuum microbiome (unconstrained PCoA ordinations analysis, A). Sample type (bulk soil, rhizosphere, and rhizoplane) presented the major driver of community variation. Ellipses are 95% confidence clouds based on standard error. Symbols refer to the different soil types. Percentage of variation given on each axis refers to the explained fraction of total variation in the fungal community. Taxonomic compositions of the top-10 fungal communities at class level (B). Fungal alpha diversity in different niches based on all samples (*n* = 16; C). Abbreviations: Bul, bulk soil; Rher, rhizosphere soil; Rlan, rhizoplane. Numbers 1, 2, 3, and 4 that follow the abbreviations represent different soil types.

Principal-coordinate analysis (PCoA) and permutational multivariate analysis of variance (PERMANOVA) analysis of the complete data set (i.e., internal transcribed spacer [ITS] sequencing data for the three compartments for each soil type) revealed that the variation in fungal community was explained mainly by the compartment niche (R^2^ = 42.1%, *P *= 0.001) and then by the soil type (R^2^ = 18.0%, *P *= 0.001) (Table 1). PCoA revealed a clear and separate clustering of the bulk soil, rhizosphere, and rhizoplane samples (Fig. 1A). We found marked differences in fungal communities when any two compartment niches were compared, with the rhizoplane-associated fungal community distinct from that in the bulk soil (R^2^ = 53.6%, *P *= 0.001) and the rhizosphere (R^2^ = 45.2%, *P *= 0.001). Furthermore, the compartment niche explained significant variations in fungal communities (R^2^ = 52% to 70%, *P *= 0.001, 0.002) in all soil types (Fig. 1A and Table 1).

**TABLE 1:** R^2^ and *P* values returned by PERMANOVA test for variance of fungal communities among different samples

Factor(s)	Results by factor		Results by niche/soil type		
	R^2^ (%)	*P* value^*a*^	Comparison^*b*^	R^2^ (%)	*P* value^*a*^
Compartment niche	42.1	**0.001**	Bul vs Rher	6.0	**0.032**
			Rher vs Rlan	45.2	**0.001**
			Bul vs Rlan	53.6	**0.001**
			Bul1 vs Rher1 vs Rlan1	66.6	**0.001**
			Bul2 vs Rher2 vs Rlan2	68.2	**0.002**
			Bul3 vs Rher3 vs Rlan3	70.2	**0.001**
			Bul4 vs Rher4 vs Rlan4	52.4	**0.002**
Soil type	18.0	**0.001**	Bul1 vs Bul2 vs Bul3 vs Bul4	52.0	**0.001**
			Rher1 vs Rher2 vs Rher3 vs Rher4	50.2	**0.001**
			Rlan1 vs Rlan2 vs Rlan3 vs Rlan4	32.9	**0.036**
Compartment niche × soil type	11.1	**0.004**			

a *P* values (based on 999 permutations) in bold indicate significant differences among tested samples (*P *< 0.05).

b Abbreviations: Bul, bulk soil; Rher, rhizosphere soil; Rlan, rhizoplane. Numbers 1, 2, 3, and 4 that follow the abbreviations represent different soil types.

Considering the compartment niche, the effect of the soil type sequentially decreased from the bulk soil (R^2^ = 52.0%, *P *= 0.001) to the rhizosphere (R^2^ = 50.2%, *P *= 0.001) to the rhizoplane (R^2^ = 32.9%, *P *= 0.036) (Table 1), and the soil chemical properties explained 68% to 74% of the fungal community variation (Table 2). The variations in the fungal community were significantly correlated (*P *< 0.005) with certain soil chemical characteristics in the bulk soil and rhizosphere communities, but the relationship was far weaker in the rhizoplane communities (*P *> 0.1). Fungal communities in the bulk soil and the rhizosphere were strongly (*P *< 0.005) correlated with soil organic material (SOM), pH, total nitrogen (TN), total potassium (TK), available phosphorus (AP), TN/total phosphorus (TP), and TN/TK (Table 2).

**TABLE 2:** Variance partition analysis and Mantel test between microbial community variance and soil chemical factors

Factor	Bulk soil			Rhizosphere soil			Rhizoplane		
	Explained^*a*^ (%)	R^*b*^	*P* value^*b*^	Explained (%)	R	*P* value	Explained (%)	R	*P* value
SOM	6.48	**0.63**	0.001	7.14	**0.70**	0.001	5.76	0.08	0.207
pH	6.65	**0.77**	0.001	6.48	**0.78**	0.001	9.43	0.00	0.456
TN	6.28	**0.76**	0.001	6.66	**0.70**	0.002	8.66	0.09	0.304
TP	6.82	0.17	0.109	7.99	0.10	0.187	6.56	0.14	0.263
TK	6.62	**0.54**	0.002	8.05	**0.50**	0.002	4.86	0.10	0.184
AP	6.96	**0.58**	0.001	6.31	**0.60**	0.002	11.59	−0.05	0.664
AK	7.11	0.13	0.152	6.52	0.20	0.076	7.53	−0.12	0.730
TN/TP	7.12	**0.57**	0.005	6.86	**0.61**	0.001	7.69	−0.03	0.371
TN/TK	7.04	**0.70**	0.001	7.39	**0.69**	0.001	6.10	0.04	0.279
TP/TK	6.89	0.20	0.094	8.00	0.23	0.059	6.31	−0.06	0.459
Residuals	32.03			28.60			25.51		

a Proportion of variance that could be explained returned by variance partition analysis.

b R and *P* values were returned by Mantel test. R values in bold means significantly correlated (*P *< 0.05, *n* = 12).

Alpha diversity analysis revealed a diversity gradient from the bulk soil to the rhizoplane; the rhizoplane communities had the lowest Chao1 (167 versus 347 and 422, *P *< 0.001) and Shannon indices (2.9 versus 6.5 and 6.9, *P *< 0.001), with an opposite pattern in the bulk soil (Fig. 1C). On the other hand, the differences in fungal alpha diversity in different soil types were marginal (see Table S3 in the supplemental material).

10.1128/msystems.00361-22.6 TABLE S3 Alpha diversity indexes of fungal communities in different compartment niches associated with all soil types. Values in table are means, and standard deviations are in parentheses (*n* = 4). Different lowercase letters (abc) represent significant differences among soil types within the same niche (*P *< 0.05; Tukey’s HSD test). Abbreviations: Bul, bulk soil; Rher, rhizosphere soil; Rlan, rhizoplane. Download Table S3, DOCX file, 0.01 MB. Copyright © 2022 Li et al. 2022 Li et al. https://creativecommons.org/licenses/by/4.0/ This content is distributed under the terms of the Creative Commons Attribution 4.0 International license.

### Changes of fungal community composition in various compartment niches.

Overall, the number of fungal taxa sequentially declined from the bulk soil to the rhizoplane compartment for all the soil types (see Fig. S1 in the supplemental material). We detected 10 fungal phyla in the bulk soil and rhizosphere compartments and 7 phyla in the rhizoplane compartment (see Table S2 in the supplemental material). The 5 dominant classes were Sordariomycetes (phylum Ascomycota), Dothideomycetes (phylum Ascomycota), Mortierellomycetes (phylum Mortierellomycota), Eurotiomycetes (phylum Ascomycota), and Agaricomycetes (phylum Basidiomycota) (Fig. 1B). However, the sequencing data indicated the existence of several deeply divergent genus-level fungal lineages that have not yet been described or sequenced previously, which were primarily enriched in the rhizoplane compartment, accounting for 91% of all total fungal genera in the rhizoplane (see Fig. S2 in the supplemental material). This finding was indicative of an insufficient database representation of the biodiversity of rhizoplane fungi.

10.1128/msystems.00361-22.1 FIG S1 The number of taxa in different compartment niches. Abbreviations: Bul, bulk soil; Rher, rhizosphere soil; Rlan, rhizoplane. Numbers 1, 2, 3, and 4 that follow the abbreviations represent different soil types. Download FIG S1, PDF file, 0.10 MB. Copyright © 2022 Li et al. 2022 Li et al. https://creativecommons.org/licenses/by/4.0/ This content is distributed under the terms of the Creative Commons Attribution 4.0 International license.

10.1128/msystems.00361-22.2 FIG S2 Heatmap displaying the relative abundances of the fungal genera that were significantly enriched or depleted in rhizoplane compared with those of bulk soil. The key from blue to red represents the least abundant to most abundant, and the numbers represent log-transformed relative abundances of the microbial community. Abbreviations: Bul, bulk soil; Rher, rhizosphere soil; Rlan, rhizoplane. Numbers 1, 2, 3, and 4 that follow the abbreviations represent different soil types. Download FIG S2, PDF file, 0.4 MB. Copyright © 2022 Li et al. 2022 Li et al. https://creativecommons.org/licenses/by/4.0/ This content is distributed under the terms of the Creative Commons Attribution 4.0 International license.

10.1128/msystems.00361-22.5 TABLE S2 Mean relative abundance (%) of the fungal phyla in different compartment niches. Data presented are means (SD); *n* = 16. Different lowercase letters indicate significant differences among compartment niches at a *P* value of <0.05 (Tukey’s HSD test). Download Table S2, DOCX file, 0.01 MB. Copyright © 2022 Li et al. 2022 Li et al. https://creativecommons.org/licenses/by/4.0/ This content is distributed under the terms of the Creative Commons Attribution 4.0 International license.

Among the identified genera, the rhizoplane compartment was significantly enriched in *Conocybe*, *Moesziomyces*, *Simplicillium*, and *unclassified_Stachybotryaceae* in comparison with the bulk soil. However, these genera were rare and with a relatively low abundance (0.01% to 0.30%) in the three compartment niches. In contrast, most of the identified fungal genera were less abundant in the rhizoplane than in the bulk soil, but the abundances of *Mortierella* and Fusarium (1.40% to 1.90%) were higher than those of other genera in the rhizoplane (Fig. S2). The distribution patterns of many of these niche-responsive genera in the three compartment niches varied with the soil type, as they were not detected in certain niches in a soil type or in a certain soil type (Fig. S2).

### Host plant selection effect reduces fungal network complexity.

**FIG 2:**
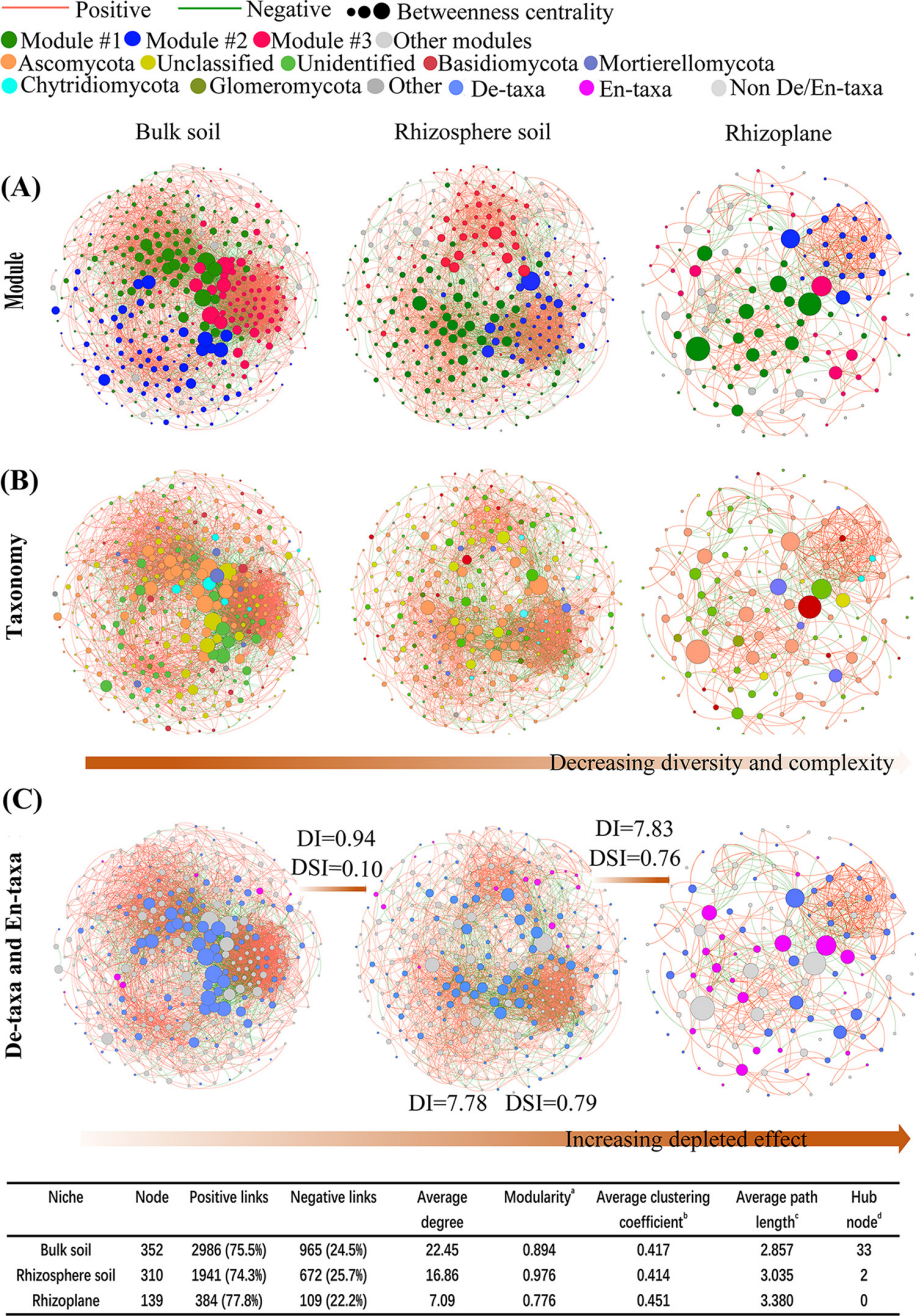
Host plant has a strong effect on reducing fungal network complexity. (A, B, C) Fungal co-occurrence networks were established along the soil-root continuum based on the ASVs table. (C) Distribution patterns of the En-taxa and De-taxa in the networks of different niches. En-taxa and De-taxa were defined as the ASV abundances that were significantly higher and lower, respectively, in rhizoplane compared with those in bulk soil compartment in this study. Red lines in the networks represent significant positive relationships, and green lines denote negative relationships (Spearman’s correlation, *P *< 0.05). The characteristics of the co-occurrence network within each compartment niche were listed in the table; the percentages in brackets represent the proportion of each number of links relative to the total links for each treatment; a, degree of nodes tending to differentiate into different network modules; b, degree of nodes tending to cluster together; c, network path distance is the length of the shortest path between two nodes within the network; d, hub node is defined as a node with high values of degree (>60) and closeness centrality (>0.4) in the network. DI, depleted index; DSI, dissimilarity index.

To further characterize the host selection effect on the fungal microbiomes, we evaluated the co-occurrence patterns of fungal communities along the soil-root continuum (Fig. 2). Fungal network complexity declined strongly along the continuum, with the highest microbial network complexity in the bulk soil (average degree, 22.45) and the lowest complexity in the rhizoplane (average degree, 7.09). Modularities of all networks were higher than 0.4, which suggests a modular network structure (41). Network modularity sequentially decreased from the rhizosphere to the bulk soil to the rhizoplane, with the modularity values 0.976 and 0.776 in the rhizosphere and rhizoplane compartments, respectively (Fig. 2A). Remarkably, the number of hub nodes notably decreased from the bulk soil to the rhizosphere to the rhizoplane. The average clustering coefficient and average path distance were highest in the rhizoplane.

The taxonomic network composition showed a similar trend in all compartments, with most nodes representing Ascomycota (41% to 48%) and Basidiomycota (5% to 6%) (Fig. 2B). The differential abundance analysis of bulk soil and rhizoplane revealed that 13.0% of amplicon sequence variants (ASVs) (26 out of 187 ASVs), mainly from the classes Sordariomycetes (5 ASVs) and unidentified_Fungi (17 ASVs), were significantly enriched in the rhizoplane (En-taxa) (see Table 3 and Table S4 in the supplemental material). In contrast, 62.6% of ASVs (117 out of 187 ASVs) were significantly depleted in the rhizoplane (De-taxa), with these ASVs mainly representing Sordariomycetes (48 ASVs) and Dothideomycetes (12 ASVs) (Fig. 2C; see Table 3 and Table S5 in the supplemental material). The hub nodes are defined commonly as taxa that exert a disproportionately large effect on the ecosystem relative to their abundance (42, 43). In the bulk soil network, 10% (12 out of 117 ASVs) of De-taxa were hub nodes (Table S5), whereas no hub nodes were identified among En-taxa in the rhizoplane network (Table S4). Of note, 3.4% (4 out of 117 ASVs) of De-taxa were important network hubs (with the highest degree value 23) in the rhizoplane (see Table S7 in the supplemental material). We used depleted index (DI) and dissimilarity index (DSI) to further evaluate taxa filtration and selection from the bulk soil to the rhizosphere and then to the rhizoplane. The DI value increased markedly, moving from the rhizosphere (0.94) to the rhizoplane (7.83), with the DSI value (0.76) of the rhizoplane higher than that of the rhizosphere (0.10). This finding demonstrates an increasing depletion from the bulk soil to the rhizosphere to the rhizoplane (Fig. 2C).

**TABLE 3:** Two sets of ASVs and the microbial taxonomy they belong to that are significantly enriched or depleted in the rhizoplane compartment compared with that of the bulk soil^*a*^

Phylum	Class	No. of ASVs	
		En-taxa	De-taxa
Ascomycota	Sordariomycetes	5	48
	Dothideomycetes	0	12
	Eurotiomycetes	0	6
	Leotiomycetes	0	2
	Saccharomycetes	0	1
	Unclassified Ascomycota	0	1
Basidiomycota	Ustilaginomycetes	1	0
	Agaricomycetes	0	1
	Malasseziomycetes	0	1
Chytridiomycota	Unidentified	0	1
Mortierellomycota	Mortierellomycetes	1	7
Glomeromycota	Paraglomeromycetes	1	0
Mucoromycota	Mucoromycetes	0	1
Unclassified Fungi	Unclassified Fungi	1	18
Unidentified	Unidentified	17	18

a Before statistics were conducted, 10% of low-variance ASVs (ASVs that were close to constant throughout the experiment conditions) were removed based on interquantile range (IQR).

10.1128/msystems.00361-22.7 TABLE S4 The set of En-taxa (26 ASVs) and the microbial taxonomy they belong to that are significantly enriched in the rhizoplane compared with those in the bulk soil compartment. Before conducting statistics, 10% of low-variance ASVs (ASVs that were close to constant throughout the experiment conditions) were removed based on interquantile range (IQR). No hub nodes (defined as a node with high values of degree [>60] and closeness centrality [>0.4]) or important network hubs were found in these En-taxa. Download Table S4, DOCX file, 0.01 MB. Copyright © 2022 Li et al. 2022 Li et al. https://creativecommons.org/licenses/by/4.0/ This content is distributed under the terms of the Creative Commons Attribution 4.0 International license.

10.1128/msystems.00361-22.8 TABLE S5 The set of De-taxa (117 ASVs) and the microbial taxonomy they belong to that are significantly depleted in the rhizoplane compared with those in the bulk soil compartment. Before conducting statistics, 10% of low-variance ASVs (ASVs that were close to constant throughout the experiment conditions) were removed based on interquantile range (IQR). Taxa in bold belonged to the hub nodes (defined as a node with high values of degree [>60] and closeness centrality [>0.4]) in the bulk soil network. The underlined taxa belonged to the important network hubs (which possessed the highest degree value of 23) in the rhizoplane network. Download Table S5, DOCX file, 0.01 MB. Copyright © 2022 Li et al. 2022 Li et al. https://creativecommons.org/licenses/by/4.0/ This content is distributed under the terms of the Creative Commons Attribution 4.0 International license.

10.1128/msystems.00361-22.10 TABLE S7 The 33 hub nodes, 2 hub nodes, and 8 important network hubs identified, respectively, in the bulk soil, rhizosphere and rhizoplane networks (at ASV level). Taxa in bold belonged to the De-taxa that were significantly depleted in rhizoplane but enriched in the bulk soil compartment. Hub node is defined as a node with high values of degree (>60) and closeness centrality (>0.4) in the network. These important network hubs possessed the highest degree value 23 in the network in the rhizoplane. Download Table S7, DOCX file, 0.01 MB. Copyright © 2022 Li et al. 2022 Li et al. https://creativecommons.org/licenses/by/4.0/ This content is distributed under the terms of the Creative Commons Attribution 4.0 International license.

### Indicator taxa in the compartment niches serve as predictors of plant agronomic traits.

**FIG 3:**
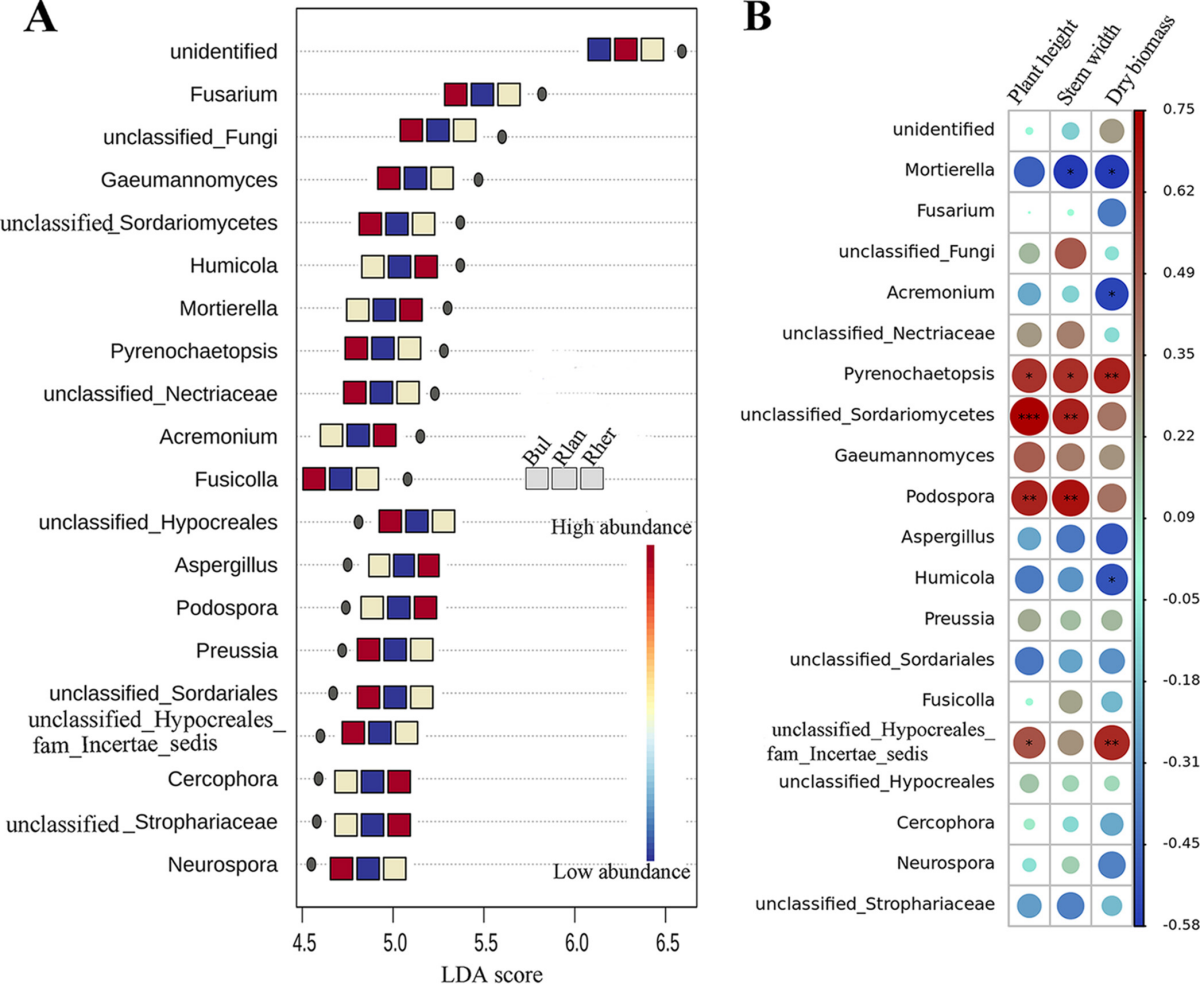
The top-20 indicator genera among the spatial compartments identified by LEfSe (A). The color of the icons shows the relative abundance of these indicators, and the key from blue to red represents the least abundant to most abundant. The icons represent bulk soil (Bul), rhizoplane (Rlan), and rhizosphere (Rher) compartments in sequence from left to right. Spearman’s correlations between the relative abundances of these indicator genera on the rhizoplane and plant agronomic traits (i.e., including plant height, stem width, and plant dry biomass) (B). The color of the icons shows the magnitude of the correlations, and the key from blue to red represents the strongest negative correlations to strongest positive correlations. *, *P* < 0.05; **, *P* < 0.01; ***, *P* < 0.001.

We used linear discriminant analysis (LDA) effect size (LEfSe) to identify the top-20 indicator genera along the soil-root continuum (Fig. 3A). Most of these genera (75%) represent to Ascomycota. Within these indicators, the unidentified genera contributed considerably (LDA score, 6.59) to fungal community variation among the compartment niches and were more abundant in the rhizoplane than in other niches. Intriguingly, all the identified genera, including Fusarium, *Gaeumannomyces*, *Humicola*, *Mortierella*, and *Pyrenochaetopsis*, were depleted in the rhizoplane in comparison with other niches. On the other hand, the indicator genera *Humicola*, *Mortierella*, *Acremonium*, Aspergillus, *Podospora*, *Cercophora*, and *unclassified_Strophariaceae* were more abundant in the rhizosphere than in other niches (Fig. 3A).

To explore how the variations in the indicator genera affect plant growth, we analyzed the correlations between the relative abundances of these indicator genera and plant agronomic traits. *Pyrenochaetopsis*, *unclassified_Sordariomycetes*, and *Podospora* (Ascomycota genera associated with the rhizoplane) were significantly (*P *< 0.05) positively correlated with plant height and stem width. *Mortierella* (Mortierellomycota) was significantly (*P *< 0.05) negatively correlated with plant stem width and dry biomass (Fig. 3B). All of these genera were among the top-20 genera identified by LEfSe and were the least abundant in the rhizoplane. Hence, we evaluated variations in ASV-level abundance in the rhizoplane compartment. Coincidentally, we identified two En-taxa belonging to *Mortierella* and *Acremonium* in the rhizoplane (Table S4), with the abundance of ASV *Mortierella* strongly correlated with all plant agronomic traits (see Fig. S3 in the supplemental material).

10.1128/msystems.00361-22.3 FIG S3 Spearman’s correlations between the relative abundances of En-taxa (that are significantly enriched in rhizoplane compartment compared with that of bulk soil) and plant agronomic traits. *, *P* < 0.05; **, *P* < 0.01. p_, phylum level; c_, class level; g_, genus level. Download FIG S3, PDF file, 0.2 MB. Copyright © 2022 Li et al. 2022 Li et al. https://creativecommons.org/licenses/by/4.0/ This content is distributed under the terms of the Creative Commons Attribution 4.0 International license.

## DISCUSSION

The plant-associated microbiome is closely related to plant growth and traits (2, 44). Few studies to date have focused on fungal community variations in the niches along the soil-root continuum, let alone simultaneously evaluated fungi in different soil types. In the present study, the pattern of fungal community separation in each niche was consistent with a spatial gradient from the bulk soil through to the rhizosphere and the rhizoplane, with similar patterns of fungi alpha diversity in each niche (Fig. 1 and Table 1). This result was in line with findings of a previous study, which demonstrated marked differences in fungal abundance patterns in the bulk soil and rhizocompartments in rice (8), indicating that the niche impacts plant fungal community assembly. However, the soil type was a lesser source of fungal community variation than the compartment niche (Table 1), with only a marginal difference in fungal alpha diversity in different soil types (Table S3). Intriguingly, although the chemical properties of different soil types were highly variable, none of the soil factors significantly affected the rhizoplane community (see Table 2 and Table S6 in the supplemental material). For instance, the effective soil properties, i.e., soil pH and nutrient stoichiometry (TN/TP, TN/TK), that fundamentally contributed to fungal community variation in the bulk soil and the rhizosphere were not associated with fungal community assembly in the rhizoplane. Hartman et al. (14) reported that fertilization drives the differences in fungal communities in the bulk soil but not in the root compartments (including the rhizoplane and endorhizosphere). Thus, the legacies of soil properties that drive the fungal community assembly are inherited by the plant rhizosphere rather than the rhizoplane, probably because the rhizoplane contains sufficient nutrients for the colonizing fungi. In addition, mutualistic associations between certain fungi and plant roots are probably involved in fungal community assembly in the rhizoplane (36, 37). These points should be addressed to allow crop microbiome manipulation in the future.

10.1128/msystems.00361-22.9 TABLE S6 Soil chemical characteristics of bulk soil of all soil types in a pot experiment. Data presented are means (SD); *n* = 3. Different lowercase letters within the same column indicate significant differences among soil types at a *P* value of <0.05 (Tukey’s HSD test). Download Table S6, DOCX file, 0.01 MB. Copyright © 2022 Li et al. 2022 Li et al. https://creativecommons.org/licenses/by/4.0/ This content is distributed under the terms of the Creative Commons Attribution 4.0 International license.

The abundance of most of the identified fungal phyla sequentially decreased from the bulk soil to the rhizoplane. Ascomycota and Basidiomycota were the most abundant phyla in the bulk soil (Table S2), broadly corresponding to the extensive surveys of soil fungal communities (45–47). On the other hand, in the current study, the abundance of Basidiomycota was lower than that of Mortierellomycota in both the rhizosphere and the rhizoplane. These observations could be explained by the notion that Basidiomycota are considered K-strategists (48), with difficulty in surviving in nutrient-abundant rhizocompartments (i.e., in comparison with the oligotrophic bulk soil) that are rich in root exudates (e.g., carbohydrates, organic acid ions, amino acids, and vitamins) (49, 50). In contrast, Mortierellomycota, Chytridiomycota, and Glomeromycota were more abundant in the rhizosphere than in the other compartment niches, indicating that the rhizosphere compartment, which is affected largely by root exudates, is the most suitable niche for these taxa, whereas they are unable to overcome the plant host immune system to successfully colonize the rhizoplane (51, 52). Indeed, in the present study, the abundance of some genera that were significantly enriched in the rhizoplane compartment was relatively low (0.01 to 0.30%) (Fig. S2).

The distinctiveness of the root-associated microbiome has been substantiated by various lines of evidence (11, 14, 53, 54). In the present study, we used the co-occurrence patterns to further examine the increasing effect of plant selection on fungal communities from the bulk soils to the rhizosphere to the rhizoplane. We recorded the highest DI and DSI values in the rhizoplane network, with the network complexity decreasing with the sequentially declining number and abundance of fungal taxa, especially those representing Ascomycota (i.e., the Ascomycota abundance decreased by 89.7%) (Fig. 2; Table 3 and S2). Similar co-occurrence patterns in the bacterial community across the above compartments have been reported previously (22). Furthermore, the higher DI value in the rhizoplane than in the rhizosphere network (7.83 versus 0.94) may indicate the attractions for some specific taxa by root exudates but a selective inhibition of their colonization of the rhizoplane by the host plant. The distribution pattern of fungal community in the rhizoplane was similar in all soil types (Fig. 1A), suggesting a considerable contribution of the host plant to the microbiota rhizoplane specialization. Niu et al. (55) reported that a simplified synthetic bacterial community reproducibly assembled on the maize root surface by incorporating the distinctive microbiota assembled by maize roots. Hence, in the present study, the depletion of complex ecological associations in fungal communities associated with the rhizoplane highlights the potential of community simplification to target their application in the plant rhizoplane. Microbiome multifunctionality in an ecosystem is positively correlated with the overall community complexity (56). Hence, the depletion of both community complexity and certain fungal taxa in the rhizoplane compartment observed in the present study is conducive to directional regulation of their target functions.

Interactions between different species determine the character of microbial community assembly and feedback effects on ecosystem function related to nutrient cycling (42, 56–59). In the current study, positive interactions (74% to 77%) between microbes dominated the co-occurrence network in the three compartments, with a higher proportion of positive interactions in the rhizoplane than those in the bulk soil or the rhizosphere (Fig. 2). Positive interactions between microbes at the same trophic level can be derived from facilitation, a process that is mutually beneficial to the interacting partners (60). Negative interactions can be an outcome of a competition for resources (61) or direct inhibition (antagonism) (62). Furthermore, in less diverse communities, fungi invest less energy in the inhibition of other microbes (e.g., by releasing secondary metabolites) (63, 64) than that in vegetative growth and secretion of decomposition enzymes (65, 66). Hence, the enhancement of facilitations among fungal taxa in the rhizoplane noted in the present study provides an opportunity to directly regulate the individual taxa to impact functional community performance. Furthermore, the interspecies relationships between fungi in the rhizoplane were closer, with a higher average clustering coefficient (0.451) than those in the bulk soil (0.417) and the rhizosphere (0.414) (Fig. 2). Collectively, these observations confirm the existence of efficient interactions between fungal taxa in the rhizoplane, which contribute to the microflora effects related to both functional features and host plant traits.

Indicator taxa play an important ecological role in microbiome assembly and ecosystem function (43, 67). The differential abundance of the top-20 indicator genera identified in the compartment niches in the present study by using LEfSe was indicative of their sensitivity to host-mediated selection (Fig. 3A). Particularly, the indicator genera *Humicola*, *Mortierella*, *Acremonium*, Aspergillus, *Podospora*, *Cercophora*, and *unclassified_Strophariaceae* were more abundant in the rhizosphere than in the other two niches (Fig. 3A), which might indicate a selective inhibition of their colonization of the rhizoplane by the host plant. In the current study, Fusarium, *Humicola*, *Mortierella*, and *Pyrenochaetopsis* accounted for much of the microbial community variation along the soil-root continuum, with Fusarium and *Mortierella* more abundant (1.40% to 1.90%) than the other indicator genera (Fig. 3A and Fig. S2). Furthermore, the relative abundances of *Mortierella* and *Pyrenochaetopsis* in the rhizoplane were good predictors of plant agronomic traits (Fig. 3B). That finding was not surprising, as *Mortierella* fungi grow rapidly on organic substrates, participate in soil nutrient cycling (68), and act as hub nodes in microbial networks for other plant species (40). Coincidently, in the present study, network hub nodes (ASV level) representing *Mortierella*, *Pyrenochaetopsis*, and *Humicola* showed high sensitivity to host plant selection in the overall community (De-taxa) (Table S7). The hub nodes are thought to exert a disproportionately large effect on the ecosystem function and microbiome assembly (43, 67). Furthermore, Fusarium fungi served as a hub node and important network hub in the bulk soil and the rhizoplane, respectively (Table S7). The high abundance of Fusarium in the soil or root frequently indicates the occurrence of plant root rot (47). Thus, the identification of these indicator taxa provides critical information for future root-associated microbiome manipulation, facilitating the application of bioinoculants for plant growth.

In conclusion, in the current study, our results demonstrated that the soil-root microbiome continuum is shaped largely by the host plant, which attracts and repels specific microbes, with a marginal influence of soil-type-dependent environmental factors. Furthermore, the depletion of complex ecological associations in the fungal community along the soil-root continuum and the enhancement of interactions among fungi in the rhizoplane provide empirical evidence for the potential of community simplification as an approach to target the plant rhizoplane for specific applications. Notably, the identified indicator taxa along the soil-root microbiome continuum provide critical information for the manipulation of the root-associated microbiome. However, sequencing data obtained in this study suggest the existence of several deeply divergent class-level fungal lineages that have not yet been described or sequenced previously, especially in the rhizoplane compartment. More efforts are needed for the continued research into fungal diversity and interactions for specific applications in the future.

## MATERIALS AND METHODS

### Preparation of experimental soils and tobacco seedlings.

Four types of experimental soils (the top 0 to 20 cm) with different soil textures were collected from tobacco fields at four different agricultural sites in Hunan Province, China, in May 2020 (see Table S1 in the supplemental material). Rice and tobacco are rotated on the sampled fields; tobacco was grown when the soils were collected. Each soil was passed through a 10-mm sieve and placed in 16- by 16- by 20-cm plastic pots for the ensuing experiments.

10.1128/msystems.00361-22.4 TABLE S1 GPS locations and textural analysis of the soil types. *Soil texture classification according to United States Department of Agriculture. Download Table S1, DOCX file, 0.01 MB. Copyright © 2022 Li et al. 2022 Li et al. https://creativecommons.org/licenses/by/4.0/ This content is distributed under the terms of the Creative Commons Attribution 4.0 International license.

Tobacco (Nicotiana tabacum L.) seeds (Yunyan 87) were provided by Yongzhou Tobacco Company, Hunan Province, China. The seeds were surface sterilized by a washing series in ethanol (2 times; 70%) and NaClO (2%). They were germinated in a mixed nursery medium composed of perlite, vermiculite, and turf (mixed in a 3:3:4 ratio). Seedlings were grown in floating polystyrene trays in a greenhouse for approximately 60 days prior to use.

### Experimental setup and sample collection.

Tobacco seedlings (described as above) were transplanted into plastic pots (2.5 kg soil per pot) at a density of 1 seedling per pot, with every soil type used in 20 pots. The pots were distributed randomly in a greenhouse (16 h light, 18/28°C night/day temperature, and 70% humidity). Plants were watered periodically every other day to maintain the maximum water retention capacity of each soil type of 55% to 65%.

Forty days after transplanting (i.e., in the vegetative phase of growth), the bulk soil, rhizosphere, and rhizoplane compartments were sampled following the procedure used by Edwards et al. (8), with modifications. Briefly, the top 1.0 cm of soil was removed, and the tobacco plants were extracted manually. The bulk soil was then sampled from the remaining soil. To collect rhizosphere samples, excess soil was shaken gently from the roots, leaving approximately 2 mm of soil still attached to the roots. The 2 mm of soil was detached directly from the roots by placing the roots in a sterile flask with 50 mL of sterile phosphate-buffered saline (PBS) and stirring vigorously. The solution was then centrifuged (4,000 × *g*, 10 min, and 4°C), with the resultant pellet used as the rhizosphere compartment. To collect rhizoplane samples, the remaining root sections were rinsed three times in PBS and then placed in a tube with 20 mL of PBS and sonicated (2 times for 30 s at 120 W and 40 kHz) to remove microbes adhering tightly to the root surface. The roots were then removed and discarded, and the rinse solution was centrifuged (4,000 × *g*, 10 min, and 4°C), with the resultant pellet used as the rhizoplane compartment. For each soil type, four biological replicates were prepared. Each replicate contained material from four pots with consistent plant agronomic traits. Soil samples were stored at −20°C until DNA extraction.

Plant height, stem width, and weight of the aboveground parts were determined. For dry biomass determination of the aboveground parts, the plants were dried in an oven at 105°C for 30 min and then incubated at 70°C for 2 days until a constant weight was achieved.

### Analysis of soil characteristics and DNA sequencing.

Chemical properties (soil organic material [SOM], total nitrogen [TN], total phosphorus [TP], total potassium [TK], available phosphorus [AP], and available potassium [AK]) of each soil type were analyzed as described by Pavan et al. (69). Soil pH was determined in aqueous suspensions of soil samples (soil/water ratio of 1:5 [wt/vol]) using a pH meter (PE-10, Sartorious, Germany).

DNA was extracted from the samples using a FastDNA spin kit for soil (MP Biomedicals, Santa Ana, CA), according to the manufacturer’s instructions. The extracted DNA was quantified, and its purity was determined based on the *A*_260_/*A*_280_ ratio, using a spectrophotometer (NanoDrop 2000; Thermo Scientific, USA). All DNA samples were diluted to 10 ng μL^−1^ and stored at −80°C for further analysis. To profile fungal communities, multiplexed barcoded ITS V1 region sequences were amplified using primers ITSF (5′-GGAAGTAAAAGTCGTAACAAGG-3′) and ITSR (5′-GCTGCGTTCTTCATCGATGC-3′). Sequencing was performed using an Illumina MiSeq platform with a paired-end protocol.

### Sequencing data processing.

Primer sequences, chimeras, and low-quality read ends with a quality score (Q) below 30 were trimmed using QIIME2 (70). DADA2 was used to infer amplicon sequence variants (ASVs). ASVs were taxonomically assigned using the UNITE (v. 8.0, unite.ut.ee) database (71). ASVs detected in only 2 samples (considered artifacts) or/and with less than 10 counts in all samples were excluded. Finally, 3,021,155 high-quality sequences of the fungal ITS region were obtained from 48 samples, with 52,958 to 67,579 sequences per sample. To correct for sampling effects, all samples were rarefied to 52,958 for downstream analyses.

### Statistical analysis.

All data were tested for normality and homogeneity of variance before further analysis of variance (ANOVA; and Tukey’s honestly significant difference [HSD] tests) to determine differences in the soil properties and fungal communities in different soil types using IBM SPSS Statistics 22.0 (SPSS Inc., Chicago, IL). Statistically significant differences were defined at a *P* value of <0.05 or the *P* values listed. ASV tables were normalized using the total sum scaling (TSS) method for beta diversity analyses.

Alpha diversity indices (Chao1, Shannon, and Goods coverage) were calculated using QIIME2. Fungal beta diversity was assessed using unconstrained principal-coordinate analysis (PCoA) based on the Bray-Curtis distance, using the “Ape” package in R (72). Permutational multivariate analysis of variance (PERMANOVA) was used to assess the effects of soil type and compartment niche and their interaction on fungal community composition with a default of 999 permutations (based on the Bray-Curtis distance using the “vegan” package in R) (73). Variation partitioning analysis (VPA) of experimental factors was performed to quantify the relative influence of soil variables on fungal community composition based on partial canonical correspondence analysis (pCCA). A Mantel test was conducted to identify correlations between fungal communities and soil variables (74).

Microbial association networks for each microcosm were created by first creating a network meta-matrix using the R package “psych” to calculate Spearman rank correlations between ASVs. The network was visualized using Gephi (v. 0.9.2) with default parameters, and the descriptive and topological indices of the networks were calculated using Gephi. Network complexity was defined as the linkage density of network nodes (i.e., average degree), according to previous studies (22, 56). Hub nodes were detected separately for each compartment niche network. The cutoffs for hub node identification were based on the distribution of the node degree (>60) and closeness centrality (>0.4) in the networks (22, 75, 76). All networks were visualized using Fruchterman-Reingold layout and both positive and negative significant correlations (*r* > 0.6, *P *< 0.05).

The differential ASV abundance in fungal communities associated with different compartment niches was evaluated using likelihood ratio test with the R package “edgeR” (77). Then, 10% of low-variance ASVs (ASVs that were close to constant throughout the experiment conditions) were removed based on interquantile range (IQR). ASVs with differing abundances in different compartment niches at a false-discovery rate-corrected *P* value of <0.05 were considered niche-responsive. En-taxa and De-taxa were defined as the ASVs with abundances that were significantly higher or lower, respectively, in the rhizoplane than those in the bulk soil compartment. Depleted index [DI = (the number of depleted ASVs)/(the number of enriched ASVs)] and dissimilarity index [DSI = (the number of depleted ASVs + the number of enriched ASVs)/(the total number of ASVs)] (22) were calculated to better understand fungal selection processes from the bulk soil to other compartment niches.

Linear discriminant analysis (LDA) effect size (LEfSe) analysis was used to perform nonparametric factorial Kruskal-Wallis (KW) sum-rank test to identify genera with significantly differential abundances in different compartment niches. This step was followed by LDA to calculate the effect size (LDA score) of each differentially abundant genus (78). Genera were considered indicator genera based on the adjusted *P* value of <0.05. Indicator genera with top-20 effect size were selected for Spearman’s correlation analysis, as these genera represent the three high abundance (>1%) phylum Ascomycota, Basidiomycota, and Mortierellomycota in the present study (Table S2).

### Data availability.

The sequences generated for this study are deposited in the NCBI Sequence Read Archive (SRA) database (accession number PRJNA818886).
