# Supplementary material for: Host Plant Selection Imprints Structure and Assembly of Fungal Community along the Soil-Root Continuum
Source: mSystems. 2022 Aug 9;7(4):e00361-22. doi: 10.1128/msystems.00361-22 (PMC9426500; doi:10.1128/msystems.00361-22)
Supplement: TABLE S3 [file msystems.00361-22-s0006.docx]

| **Diversity indexes** | **Soil1** | | |  | **Soil2** | | |  | **Soil3** | | |  | **Soil4** | | |
| --- | --- | --- | --- | --- | --- | --- | --- | --- | --- | --- | --- | --- | --- | --- | --- |
|  | **Bul** | **Rher** | **Rlan** |  | **Bul** | **Rher** | **Rlan** |  | **Bul** | **Rher** | **Rlan** |  | **Bul** | **Rher** | **Rlan** |
| Chao 1 | 476.05  (65.91) | 371.97  (59.64) | 220.13  (13.88)**b** |  | 410.62  (37.60) | 316.49  (33.02) | 149.37  (28.77)**a** |  | 356.43  (40.52) | 355.00  (91.68) | 140.08  (37.45)**a** |  | 442.98  (94.83) | 345.94  (47.85) | 157.66  (17.44)**a** |
| Goods coverage (‰) | 999.867  (0.02) | 999.93  (0.01) | 999.94  (0.01) |  | 999.87  (0.03) | 999.90  (0.03) | 999.96  (0.03) |  | 999.88  (0.03) | 999.86  (0.08) | 999.97  (0.01) |  | 999.88  (0.05) | 999.93  (0.03) | 999.97  (0.03) |
| Shannon | 7.09  (0.23)**ab** | 6.72  (0.13) | 2.98  (0.20) |  | 7.10  (0.20)**b** | 6.41  (0.30) | 2.66  (0.39) |  | 6.52  (0.16)**a** | 6.56  (0.18) | 2.62  (0.34) |  | 6.90  (0.43)**ab** | 6.17  (0.72) | 3.37  (0.89) |
