# Supplementary figures and images for: Host Plant Selection Imprints Structure and Assembly of Fungal Community along the Soil-Root Continuum
Source: mSystems. 2022 Aug 9;7(4):e00361-22. doi: 10.1128/msystems.00361-22 (PMC9426500; doi:10.1128/msystems.00361-22)

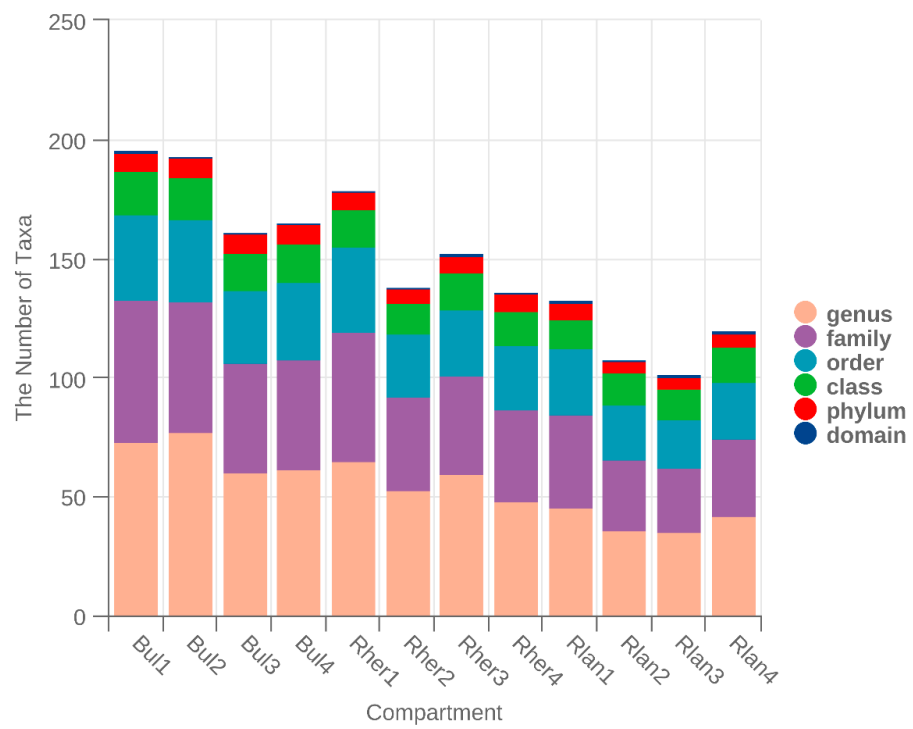

Supplement: FIG S1 [file msystems.00361-22-s0001.pdf]

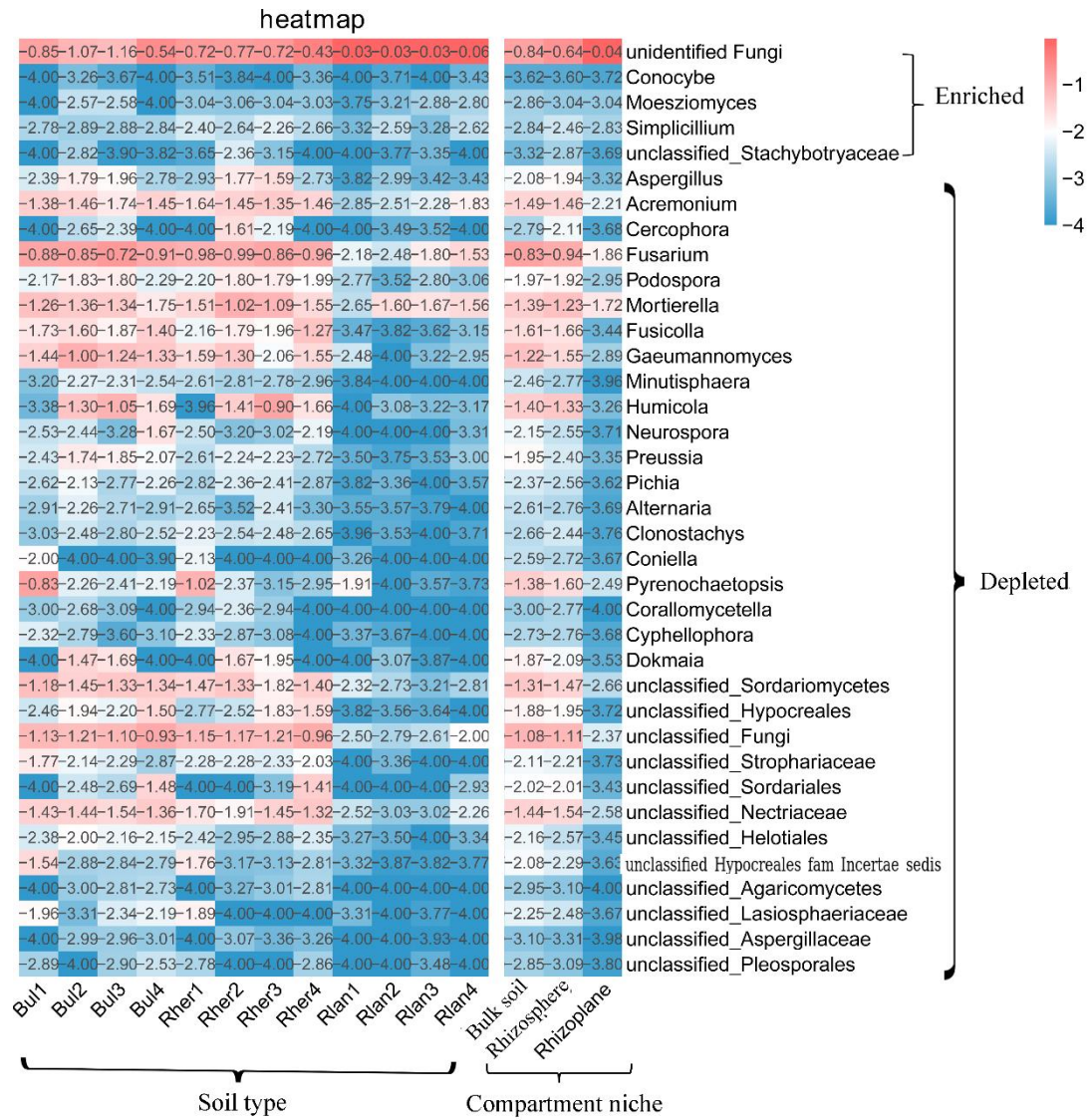

Supplement: FIG S2 [file msystems.00361-22-s0002.pdf]

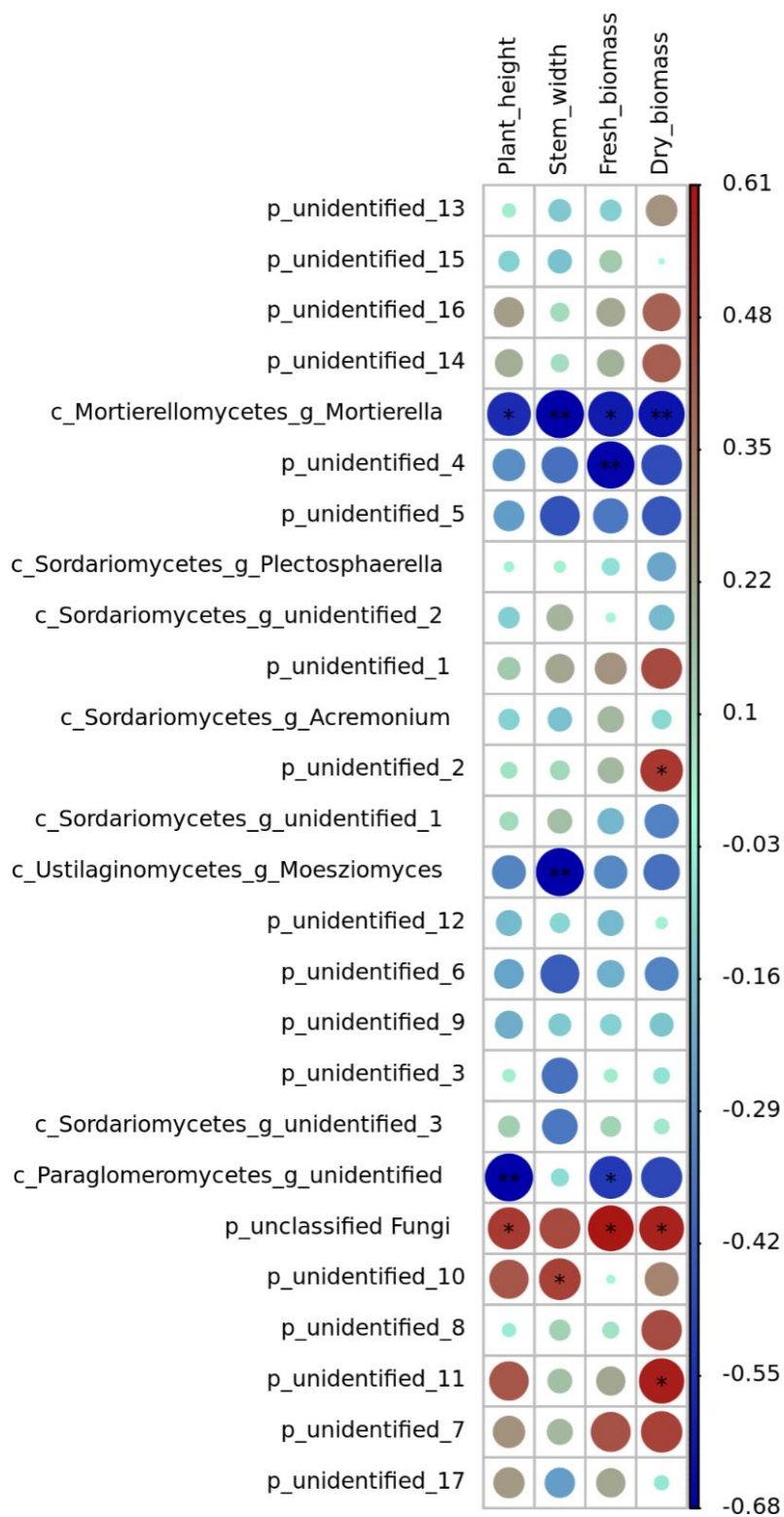

Supplement: FIG S3 [file msystems.00361-22-s0003.pdf]
