# Supplementary material for: Host Plant Selection Imprints Structure and Assembly of Fungal Community along the Soil-Root Continuum
Source: mSystems. 2022 Aug 9;7(4):e00361-22. doi: 10.1128/msystems.00361-22 (PMC9426500; doi:10.1128/msystems.00361-22)
Supplement: TABLE S2 [file msystems.00361-22-s0005.docx]

| **Phylum** | **Bulk soil (%)** | **rhizosphere soil (%)** | **Rhizoplane (%)** |
| --- | --- | --- | --- |
| Ascomycota | 63.89 (10.27)b | 58.71 (12.18)b | 6.55 (6.03)a |
| Basidiomycota | 3.87 (1.59)b | 2.87 (1.29)b | 0.74 (0.68)a |
| Mortierellomycota | 3.37 (1.52)ab | 4.78 (3.66)b | 1.86 (2.22)a |
| Chytridiomycota | 1.11 (1.64) | 1.36 (3.20) | 0.45 (1.52) |
| Glomeromycota | 0.47 (0.39)b | 0.54 (0.42)b | 0.12 (0.18)a |
| Rozellomycota | 0.18 (0.41) | 0.05 (0.14) | **0.00** (0.00) |
| Aphelidiomycota | 0.20 (0.57) | 0.03 (0.13) | **0.00** (0.00) |
| Blastocladiomycota | 0.09 (0.13)b | 0.04 (0.11)ab | **0.00** (0.00)a |
| Mucoromycota | 0.10 (0.16) | 0.09 (0.13) | 0.01 (0.02) |
| Olpidiomycota | 0.08 (0.13) | 0.06 (0.13) | 0.01 (0.03) |
| unclassified_Fungi | 16.46 (4.46)c | 13.10 (3.70)b | 0.83 (0.65)a |
| unidentified | 10.18 (11.46)a | 18.35 (14.16)a | 89.41 (8.37)b |
