# Supplementary material for: Host Plant Selection Imprints Structure and Assembly of Fungal Community along the Soil-Root Continuum
Source: mSystems. 2022 Aug 9;7(4):e00361-22. doi: 10.1128/msystems.00361-22 (PMC9426500; doi:10.1128/msystems.00361-22)
Supplement: TABLE S4 [file msystems.00361-22-s0007.docx]

| **Phylum** | **Class** | **Genus** | **ASVs counts** | **ASV ID** |
| --- | --- | --- | --- | --- |
| unidentified Fungi | unidentified Fungi | *Unidentified Fungi* | 17 | ASV_3958, ASV_6064, ASV_4130, ASV_3886, ASV_2006, ASV_2843, ASV_3678, ASV_2055, ASV_5868, ASV_3380, ASV_5943, ASV_5728, ASV_2733, ASV_3692, ASV_3211, ASV_4251, ASV_2932 |
| Mortierellomycota | Mortierellomycetes | *Mortierella* | 1 | ASV_2289 |
| Ascomycota | Sordariomycetes | *unidentified* | 3 | ASV_4282, ASV_2214, ASV_1346 |
|  |  | *Acremonium* | 1 | ASV_5120 |
|  |  | *Plectosphaerella* | 1 | ASV_3731 |
| unclassified Fungi | unclassified Fungi | *unclassified Fungi* | 1 | ASV_3295 |
| Glomeromycota | Paraglomeromycetes | *unidentified* | 1 | ASV_6372 |
| Basidiomycota | Ustilaginomycetes | *Moesziomyces* | 1 | ASV_2199 |
