# Supplementary material for: Host Plant Selection Imprints Structure and Assembly of Fungal Community along the Soil-Root Continuum
Source: mSystems. 2022 Aug 9;7(4):e00361-22. doi: 10.1128/msystems.00361-22 (PMC9426500; doi:10.1128/msystems.00361-22)
Supplement: TABLE S5 [file msystems.00361-22-s0008.docx]

| **Phylum** | **Class** | **Genus (counts of ASVs)** | **ASV ID** |
| --- | --- | --- | --- |
| Ascomycota | Sordariomycetes | *unclassified Hypocreales* (3); *Acremonium* (5); *Apodus* (1); *Botryotrichum* (1); *Cercophora* (1); ***Chaetomium*** (1); *Clonostachys* (2); ***Coniella*** (1); ***Coniochaeta*** (1); *Corallomycetella* (1); *Fusarium* (4); *Fusicolla* (1); *Gaeumannomyces* (1); ***Humicola*** (3); *Lecythophora* (1); *Lophotrichus* (1); *Metarhizium* (1); *Mycothermus* (1); *Neurospora* (1); *Plectosphaerella* (1); *Podospora* (1); *Schizothecium* (1); ***Trichoderma*** (1); *unclassified* *Nectriaceae* (3); *unclassified Sordariales* (1); *unclassified Sordariomycetes* (5); ***unidentified*** (4) | ASV_121,ASV_4632,ASV_3849; ASV_2939,ASV_1899,ASV_4859,ASV_1525,ASV_2213; ASV_3497; ASV_3076; ASV_1981; **ASV_1570**; ASV_5941,ASV_3879; **ASV_4964**; **ASV_595**; ASV_1330; ASV_5172,ASV_2937,ASV_5302,ASV_5434; ASV_6374; ASV_3360; ASV_5887,ASV_5341,**ASV_3117**; ASV_1503; ASV_5813; ASV_2597; ASV_5850; ASV_2724; ASV_471; ASV_2363; ASV_5901; **ASV_5834**; ASV_5426,ASV_1085,ASV_23; ASV_4377; ASV_1650,ASV_2700,ASV_3663,ASV_483,ASV_6643; ASV_2202,ASV_2202,**ASV_6379**, **ASV_4703** |
|  | Dothideomycetes | *Alternaria* (1); *Cladosporium* (1); *Curvularia* (1); *Didymella* (1); *Dokmaia* (1); *Minutisphaera* (1); *Mycosphaerella* (1); *Preussia* (1); ***Pyrenochaetopsis*** (3); *unclassified Pleosporales* (1) | ASV_3808; ASV_2769; ASV_6712; ASV_400; ASV_3520; ASV_6193; ASV_1347; ASV_5804; **ASV_1033**, ASV_3433, ASV_5697; ASV_424 |
|  | Eurotiomycetes | *Aspergillus* (2); *Cyphellophora* (1); ***Talaromyces*** (2); *unclassified Aspergillaceae* (1) | ASV_835, ASV_5481; ASV_1011; ASV_1434, **ASV_6213**; ASV_5840 |
|  | Leotiomycetes | *unclassified Helotiales* (2) | ASV_3032, ASV_2753 |
|  | Saccharomycetes | *Pichia* (1) | ASV_4926 |
|  | Unclassified Ascomycota | *unclassified Ascomycota* (1) | ASV_2416 |
| Basidiomycota | Agaricomycetes | *unclassified Strophariaceae* (1) | ASV_4690 |
|  | Malasseziomycetes | *unidentified* (1) | ASV_1927 |
| Chytridiomycota | unidentified | ***unidentified*** (1) | **ASV_4542** |
| Mortierellomycota | Mortierellomycetes | ***Mortierella*** (7) | ASV_5772, ASV_3354, ASV_381, ASV_1984,**ASV_4650**,ASV_5136, ASV_5844 |
| Mucoromycota | Mucoromycetes | *Rhizopus* (1) | ASV_6074 |
| unclassified Fungi | unclassified Fungi | *unclassified Fungi* (18) | ASV_687, ASV_3466, ASV_811, ASV_986, ASV_1470, ASV_5757, ASV_3592,ASV_4550,ASV_1054,ASV_5624,ASV_3368, ASV_957, ASV_3088,ASV_6331,ASV_4620,ASV_4579,ASV_3477,ASV_2903, |
| unidentified | unidentified | ***unidentified*** (18) | ASV_5310,ASV_6132,ASV_2466,ASV_5188,**ASV_5783**, ASV_351, ASV_2127, ASV_3860, ASV_415, ASV_4906,ASV_4535,ASV_3044,ASV_6554,ASV_448, ASV_4319, ASV_5843,ASV_3278, ASV_3417 |
