# Supplementary material for: Host Plant Selection Imprints Structure and Assembly of Fungal Community along the Soil-Root Continuum
Source: mSystems. 2022 Aug 9;7(4):e00361-22. doi: 10.1128/msystems.00361-22 (PMC9426500; doi:10.1128/msystems.00361-22)
Supplement: TABLE S7 [file msystems.00361-22-s0010.docx]

| **Compartment** | **Phylum** | **Class** | **Genus** | **ASV ID** |
| --- | --- | --- | --- | --- |
|  | Ascomycota | Dothideomycetes | ***Pyrenochaetopsis*** | ASV_1033 |
|  |  | Eurotiomycetes | *Aspergillus* | ASV_4710 |
|  |  |  | *Exophiala* | ASV_2832 |
|  |  |  | ***Talaromyces*** | ASV_6213 |
|  |  | Saccharomycetes | *Candida* | ASV_1664 |
|  |  |  | ***Chaetomium*** | ASV_1570 |
|  |  |  | ***Coniella*** | ASV_4964 |
|  |  |  | ***Coniochaeta*** | ASV_595 |
|  |  |  | *Fusarium* | ASV_3462 |
|  |  |  | ***Humicola*** | ASV_3117 |
|  |  |  | ***Trichoderma*** | ASV_5834 |
|  |  |  | *unclassified_Lasiosphaeriaceae* | ASV_6312 |
|  |  |  | ***Unidentified_1*** | ASV_6379 |
|  |  |  | *Unidentified_2* | ASV_4702 |
|  |  |  | ***Unidentified_3*** | ASV_4703 |
|  | Basidiomycota | unclassified_Basidiomycota | *Unclassified_Basidiomycota* | ASV_4938 |
| **Bulk soil** | Chytridiomycota | Rhizophlyctidomycetes | *Rhizophlyctis* | ASV_4175 |
|  |  | Unidentified_1 | *Unidentified_1* | ASV_6605 |
|  |  | Unidentified_2 | ***Unidentified_2*** | ASV_4542 |
|  | Mortierellomycota | Mortierellomycetes | ***Mortierella*** | ASV_4650 |
|  | unclassified Fungi_1 | unclassified Fungi_1 | *unclassified Fungi_1* | ASV_127 |
|  | unclassified Fungi_2 | unclassified Fungi_2 | *unclassified Fungi_2* | ASV_2295 |
|  | unclassified Fungi_3 | unclassified Fungi_3 | *unclassified Fungi_3* | ASV_4233 |
|  | unclassified Fungi_4 | unclassified Fungi_4 | *unclassified Fungi_4* | ASV_2715 |
|  | unclassified Fungi_5 | unclassified Fungi_5 | *unclassified Fungi_5* | ASV_3340 |
|  | unclassified Fungi_6 | unclassified Fungi_6 | *unclassified Fungi_6* | ASV_634 |
|  | Unidentified_1 | Unidentified_1 | *Unidentified_1* | ASV_3136 |
|  | Unidentified_2 | Unidentified_2 | *Unidentified_2* | ASV_1047 |
|  | Unidentified_3 | Unidentified_3 | *Unidentified_3* | ASV_1147 |
|  | Unidentified_4 | Unidentified_4 | *Unidentified_4* | ASV_2776 |
|  | Unidentified_5 | Unidentified_5 | *Unidentified_5* | ASV_3823 |
|  | Unidentified_6 | Unidentified_6 | ***Unidentified_6*** | ASV_5783 |
|  | Unidentified_7 | Unidentified_7 | *Unidentified_7* | ASV_6068 |
| **Rhizosphere** | Ascomycota | Sordariomycetes | ***Lecythophora*** | ASV_1503 |
|  |  | Eurotiomycetes | *Talaromyces* | ASV_4309 |
|  | Ascomycota | Sordariomycetes | ***Fusarium*** | ASV_2937 |
|  |  |  | *Lecanicillium* | ASV_671 |
|  |  |  | ***Metarhizium*** | ASV_2597 |
| **Rhizoplane** |  |  | ***Podospora*** | ASV_2363 |
|  |  |  | ***unidentified*** | ASV_6379 |
|  | Basidiomycota | Agaricomycetes | *Thanatephorus* | ASV_248 |
|  | Chytridiomycota | Rhizophlyctidomycetes | *Rhizophlyctis* | ASV_4175 |
|  | Chytridiomycota | Rhizophlyctidomycetes | *Rhizophlyctis* | ASV_3997 |
