# Supplementary material for: Host Plant Selection Imprints Structure and Assembly of Fungal Community along the Soil-Root Continuum
Source: mSystems. 2022 Aug 9;7(4):e00361-22. doi: 10.1128/msystems.00361-22 (PMC9426500; doi:10.1128/msystems.00361-22)
Supplement: TABLE S6 [file msystems.00361-22-s0009.docx]

| **Soil type** | **pH** | **AP mg·kg^-1^** | **AK mg·kg^-1^** | **TN g·kg^-1^** | **TP g·kg^-1^** | **TK g·kg^-1^** | **SOM g·kg^-1^** | **TN/TP** | **TN/TK** | **TP/TK** |
| --- | --- | --- | --- | --- | --- | --- | --- | --- | --- | --- |
| Soil1 | 6.91  (0.01)a | 19.74  (0.38)a | 225.69  (14.42)a | 2.74  (0.03)c | 1.46  (0.10) | 8.83  (0.54)a | 50.45  (4.19)b | 1.88  (0.15)b | 0.31  (0.02)b | 0.17  (0.01)ab |
| Soil2 | 7.49  (0.02)d | 34.49  (0.85)b | 236.91  (20.66)a | 2.24  (0.07)b | 1.52  (0.05) | 10.03  (0.03)b | 40.65  (1.25)a | 1.47  (0.02)a | 0.22  (0.01)a | 0.15  (0.00)a |
| Soil3 | 7.36  (0.05)c | 39.13  (2.01)c | 313.05  (14.56)b | 2.34  (0.07)b | 1.58  (0.07) | 10.20  (0.20)b | 39.63  (2.26)a | 1.48  (0.09)a | 0.23  (0.00)a | 0.15  (0.01)ab |
| Soil4 | 7.17  (0.03)b | 21.34  (1.08)a | 279.66  (23.23)b | 2.04  (0.04)a | 1.43  (0.06) | 8.36  (0.13)a | 35.35  (0.45)a | 1.43  (0.06)a | 0.24  (0.01)a | 0.17  (0.01)b |
