# Supplementary material for: Host Plant Selection Imprints Structure and Assembly of Fungal Community along the Soil-Root Continuum
Source: mSystems. 2022 Aug 9;7(4):e00361-22. doi: 10.1128/msystems.00361-22 (PMC9426500; doi:10.1128/msystems.00361-22)
Supplement: TABLE S1 [file msystems.00361-22-s0004.docx]

| **Soil type** | **GPS location** | **Distribution of soil particle size** | | | **Classification*** |
| --- | --- | --- | --- | --- | --- |
|  |  | **Sand (%)** | **Silt (%)** | **Clay (%)** |  |
| Soil1 | 112.240731 E/25.523955 N | 11.36 | 63.36 | 25.28 | silt loam |
| Soil2 | 111.511822 E/24.819746N | 39.76 | 40.28 | 19.96 | loam |
| Soil3 | 111.600866 E/25.526282 N | 19.92 | 44.84 | 35.20 | silty clay loam |
| Soil4 | 112.254135 E/25.525936 N | 12.24 | 44.72 | 43.04 | silty clay |
